# Supplementary material for: Microglia play beneficial roles in multiple experimental seizure models
Source: bioRxiv. 2023 Mar 6:2023.03.04.531090. Preprint. [Version 1] doi: 10.1101/2023.03.04.531090 (PMC10028974; doi:10.1101/2023.03.04.531090)
Supplement: Supplement 1 [file NIHPP2023.03.04.531090v1-supplement-1.pdf]

bioRxiv preprint doi: https://doi.org/10.1101/2023.03.04.531090; this version posted March 6, 2023. The copyright holder for this preprint (which was not certified by peer review) is the author/funder, who has granted bioRxiv a license to display the preprint in perpetuity. It is made available under aCC-BY 4.0 International license.

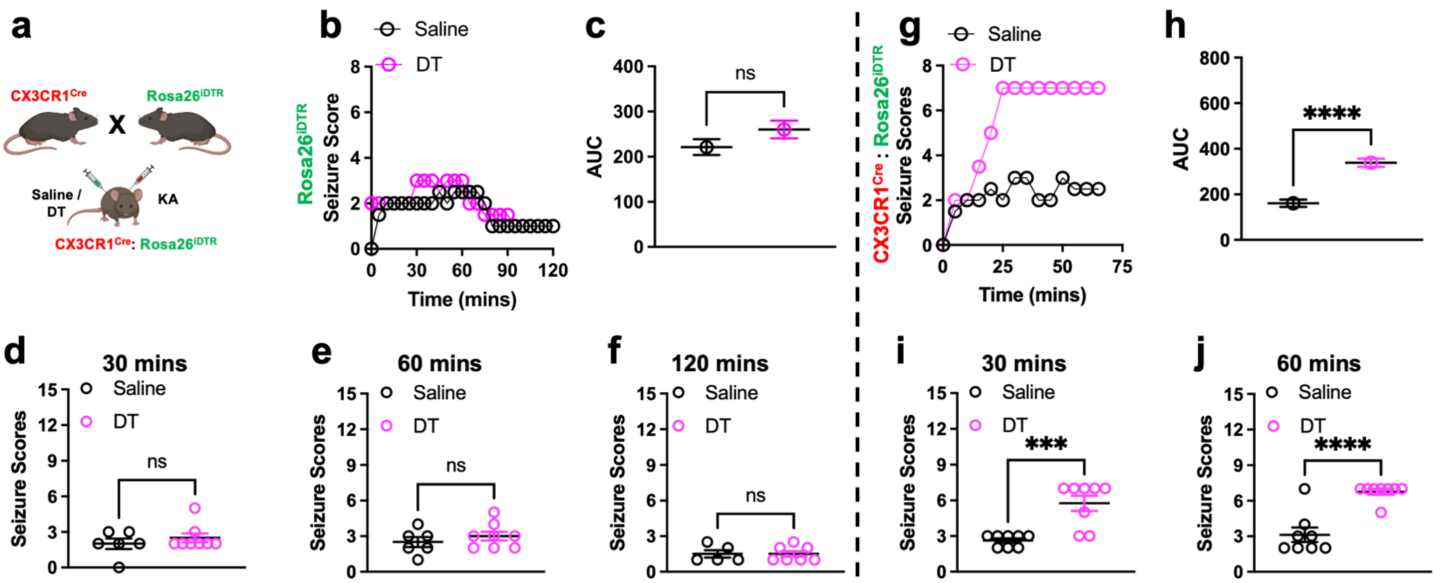

SUPPLEMENTAL FIGURE 1. Pharmacogenetic microglial elimination and chemoconvulsive seizures. (a) Mating scheme of mice generation and experimental setup. (b and c) Overall (b) and average (c) Racine seizure scores from saline or diphtheria toxin (DT) treated Rosa26^{iDTR}. n = 6-8 mice per group (d and e) Overall (d) and average (e) Racine seizure scores from saline or DT treated CX3CR1^{Cre}: Rosa26^{iDTR} mice. n = 8 mice per group. Data presented as median in b and d and mean ± s.e.m in c and e. Statistics calculated by Student's T-test, ****p < 0.0001.

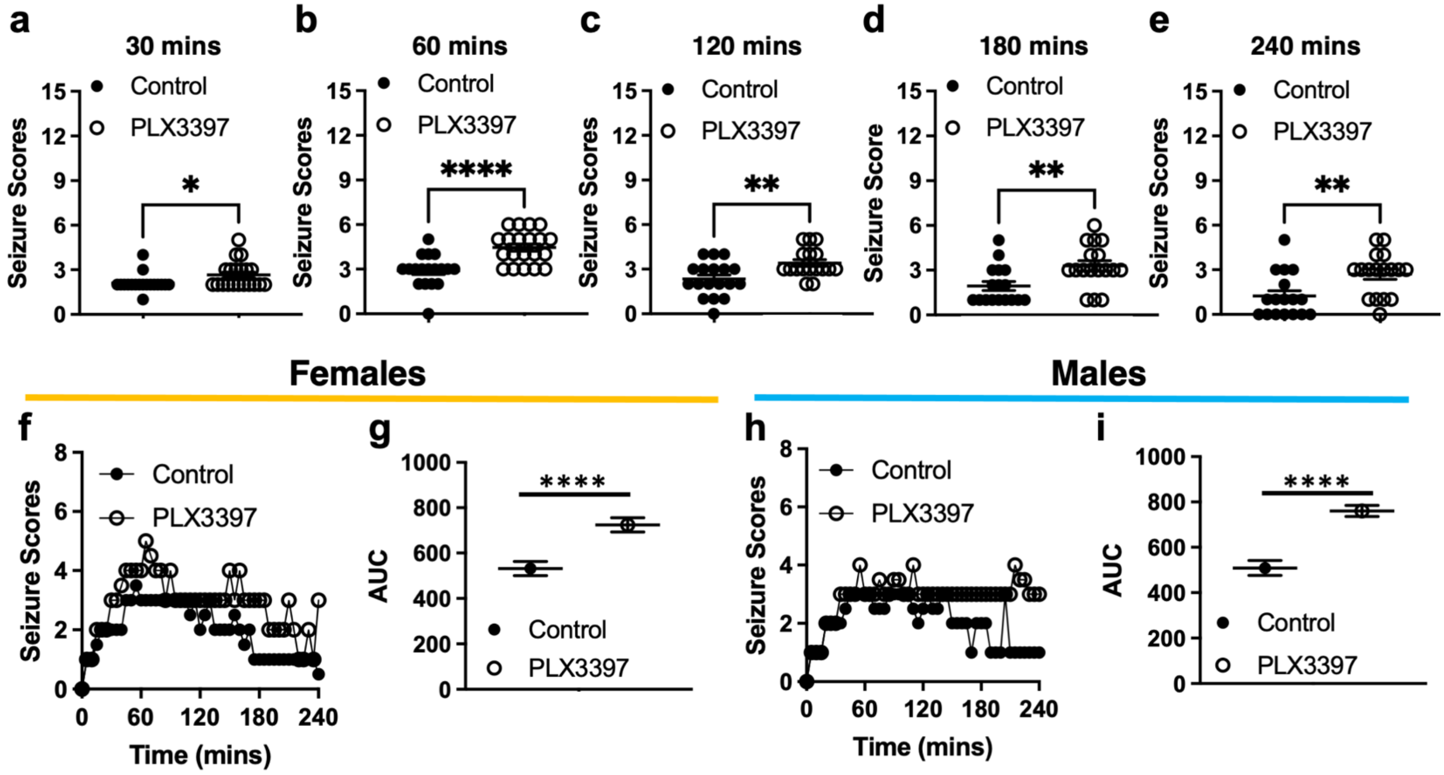

SUPPLEMENTAL FIGURE 2. Time and sex features of chemoconvulsive kainic acid-induced seizures with microglial pharmacological elimination. (a- e) Racine seizure scores at various time points from control and PLX3397 treated mice over 240 mins. n = 18-20 mice per group. (f - i) Overall (f, h) and area under the curve (AUC, g, i) Racine seizure scores from control and PLX3397 treated female (f – g) and male (h – i) mic. Data presented as mean ± s.e.m in a – e, g and i and as median in f and h. Statistics calculated by Student's T-test, *p < 0.05, **p < 0.01; ****p < 0.0001.

bioRxiv preprint doi: https://doi.org/10.1101/2023.03.04.531090; this version posted March 6, 2023. The copyright holder for this preprint (which was not certified by peer review) is the author/funder, who has granted bioRxiv a license to display the preprint in perpetuity. It is made available under aCC-BY 4.0 International license.

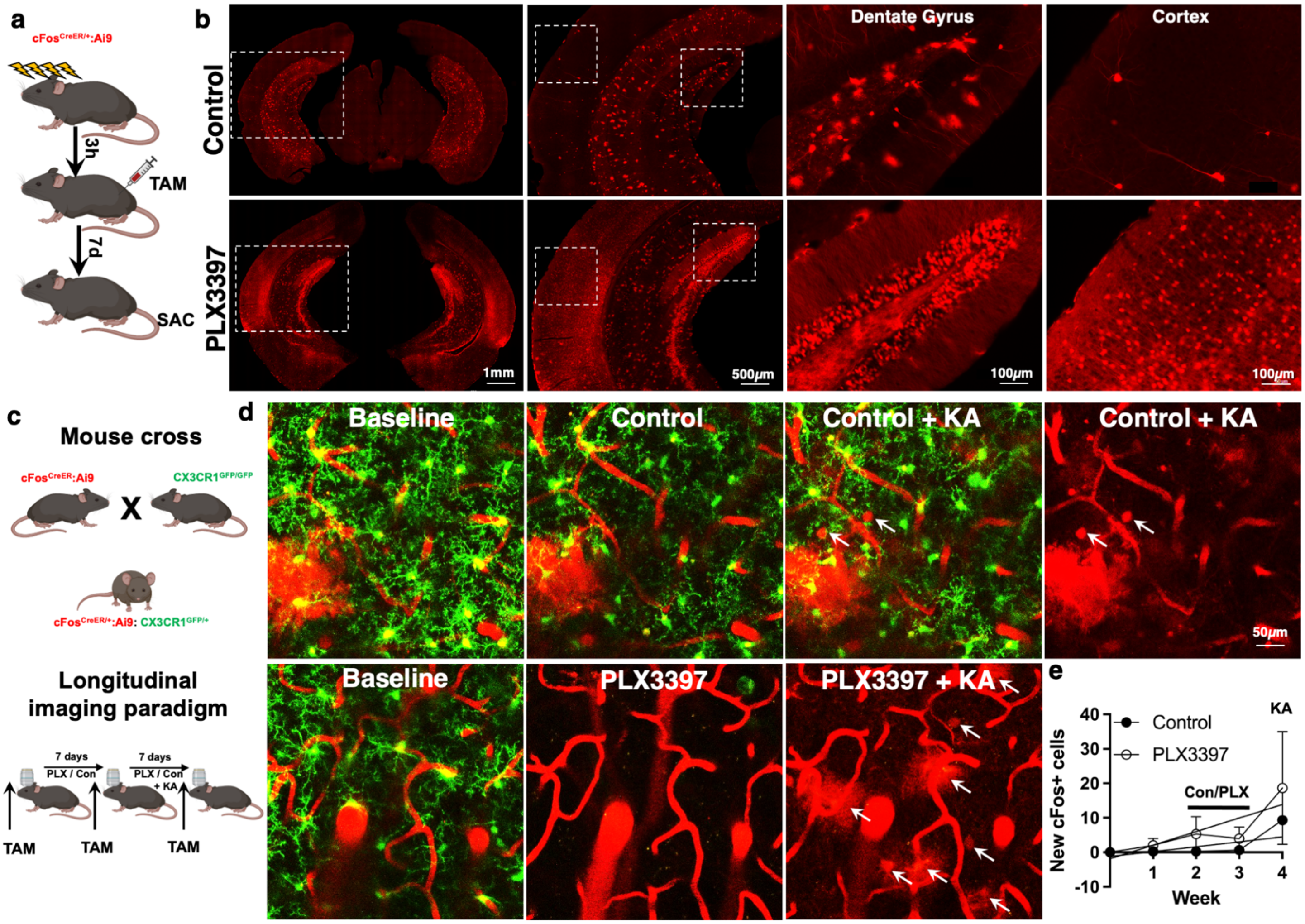

SUPPLEMENTAL FIGURE 3. Pharmacological microglial elimination increases brain activity. (a) Experimental protocol for seizure cFos cell labelling following KA-induced seizures (b) Representative images of tdTomato-expression under the cFos promoter in cFos^{tdTomato} mice under baseline conditions and at 3h of KA after a 7-day treatment of control of PLX3397 in the cortex and hippocampus. (c) Experimental cross (top) and scheme for monitoring protocol for seizure cFos cell labelling following KA-induced seizures (bottom). (d) Representative longitudinal *in vivo* two-photon images of microglial (green), blood vessels (tubular red structures), and cFos positive (red circular structures) in different conditions. (e) Quantification of the average intensity of tdTomato fluorescence in the different conditions with longitudinal *in vivo* two-photon imaging. Mice were monitored for 2 weeks without PLX3397 and then for two weeks with control or PLX3397 chow before treatment with KA. n = 2 mice per group. Data presented as mean ± s.e.m.

bioRxiv preprint doi: https://doi.org/10.1101/2023.03.04.531090; this version posted March 6, 2023. The copyright holder for this preprint (which was not certified by peer review) is the author/funder, who has granted bioRxiv a license to display the preprint in perpetuity. It is made available under aCC-BY 4.0 International license.

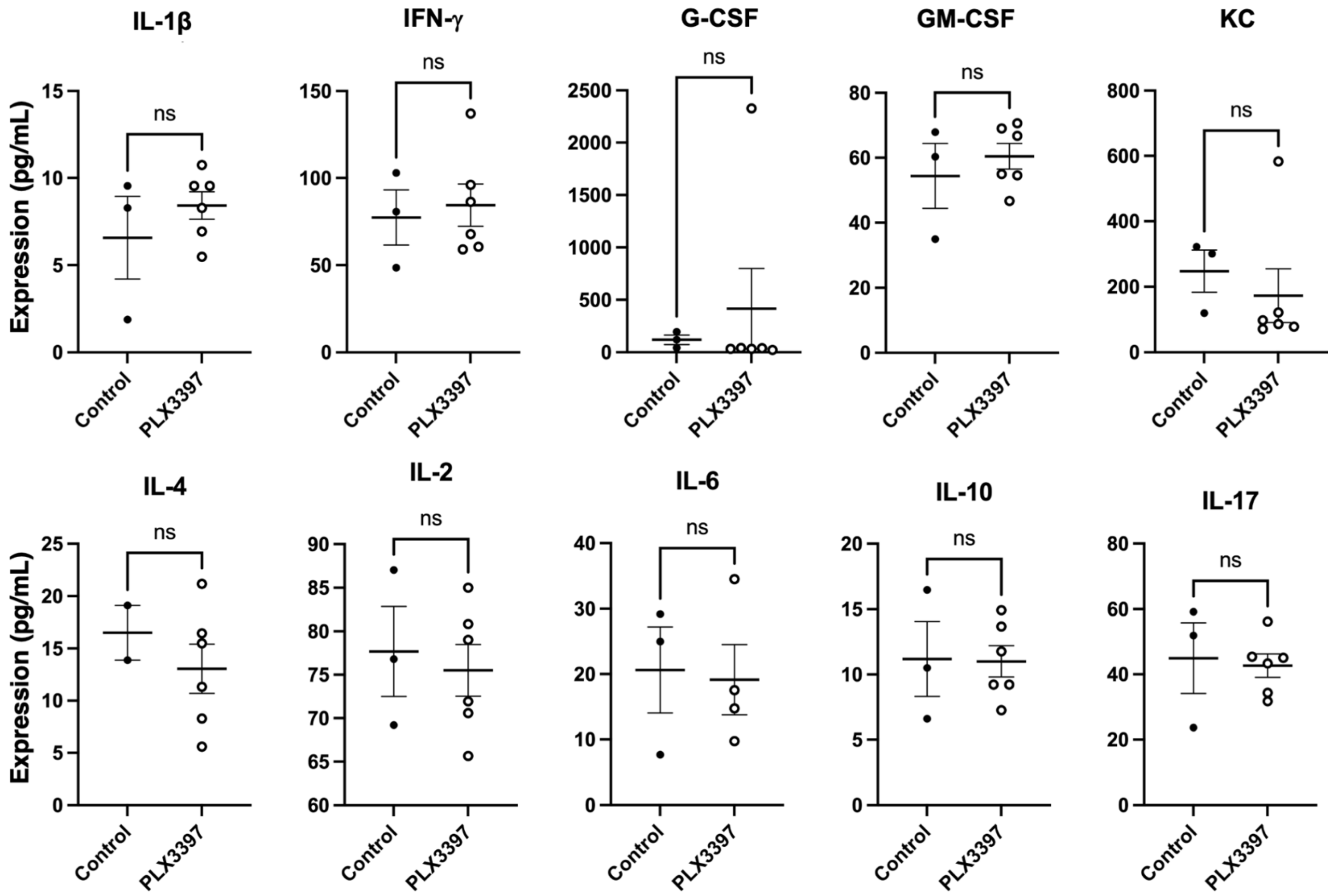

SUPPLEMENTAL FIGURE 4. Microglial elimination does not alter cytokine levels. (Expression levels for various cytokines from control and PLX3397-treated brains after a week of treatment. n = 2-5 mice per group. Statistics calculated by Student's T-test. Data presented as mean ± s.e.m.

bioRxiv preprint doi: https://doi.org/10.1101/2023.03.04.531090; this version posted March 6, 2023. The copyright holder for this preprint (which was not certified by peer review) is the author/funder, who has granted bioRxiv a license to display the preprint in perpetuity. It is made available under aCC-BY 4.0 International license.

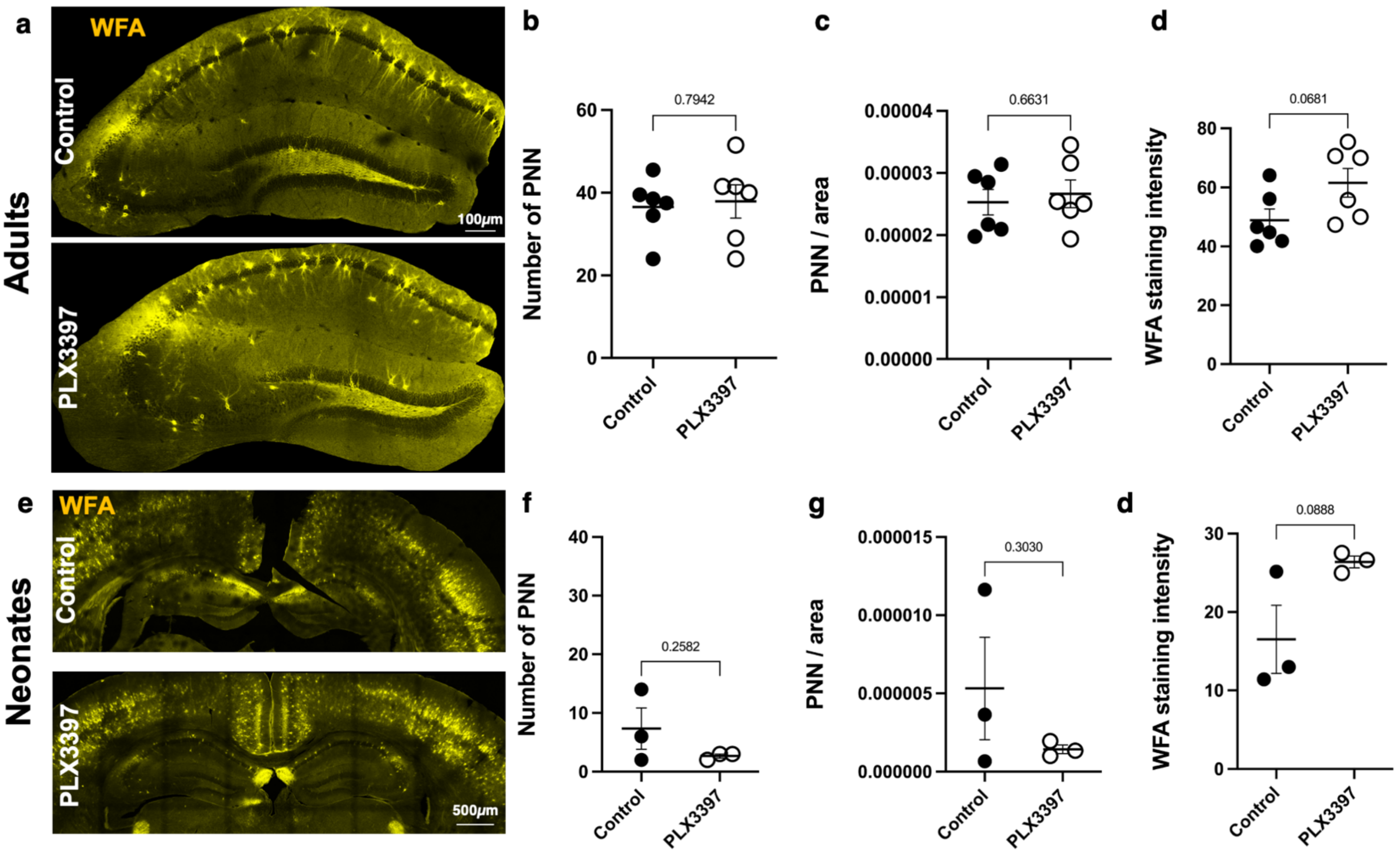

SUPPLEMENTAL FIGURE 5. Microglial elimination does not alter perineuronal net density. (a) Representative images of hippocampal tissues stained with WFA (yellow) to label perineuronal nets (PNNs) from control and PLX3397-treated adult brains after a week of treatment. (b - d) Quantification of the number of PNN (WFA$^+$) cells (b), the area occupied by PNN (WFA$^+$) in hippocampal tissue (c), and the fluorescence intensity of WFA (d) from control and PLX3397-treated brains after a week of treatment. n = 6 mice per group. (e) Representative images of hippocampal tissues stained with WFA (yellow) to label perineuronal nets (PNNs) from control and PLX3397-treated neonatal brains after 2 days of treatment. (f - h) Quantification of the number of PNN (WFA$^+$) cells (f), the area occupied by PNN (WFA$^+$) in hippocampal tissue (g), and the fluorescence intensity of WFA (h) from control and PLX3397-treated brains after 2 days of treatment. n = 3 mice per group. Statistics calculated by Student's T-test. Data presented as mean ± s.e.m.

bioRxiv preprint doi: https://doi.org/10.1101/2023.03.04.531090; this version posted March 6, 2023. The copyright holder for this preprint (which was not certified by peer review) is the author/funder, who has granted bioRxiv a license to display the preprint in perpetuity. It is made available under aCC-BY 4.0 International license.

SUPPLEMENTAL VIDEOS AND LEGENDS

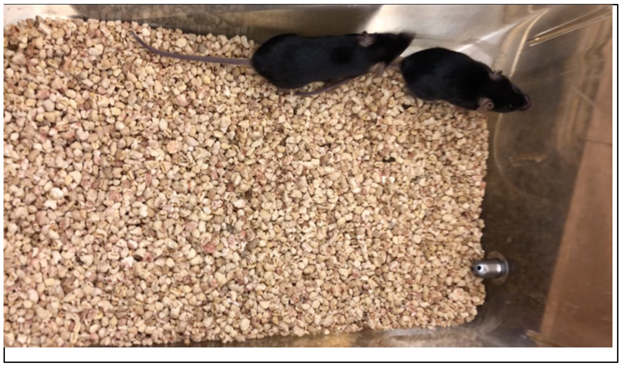

SUPPLEMENTAL VIDEO S1. Mouse activity at 2 days of KA treatment in control mice. Wildtype mice exploring their cage at 2 days after experiencing KA-induced status epilepticus. Mice are actively exploring their environment. The movie is 15 seconds long.

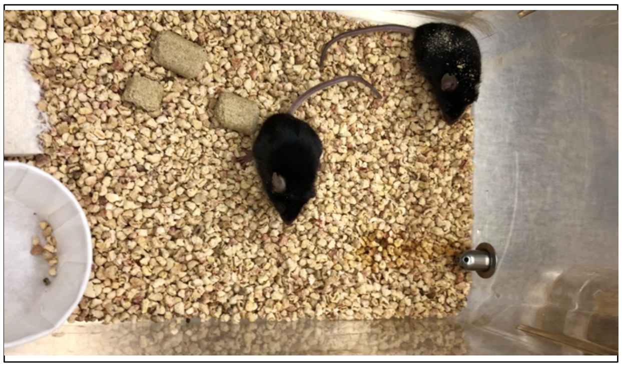

SUPPLEMENTAL VIDEO S2. Mouse activity at 2 days of KA treatment in PLX3397-treated mice. Wildtype mice were exposed to PLX3397 for 7 days and then treated with KA. This movie is collected at 2 days after KA-induced status epilepticus and mice are not as active in exploring their cage. The movie is 16 seconds long.

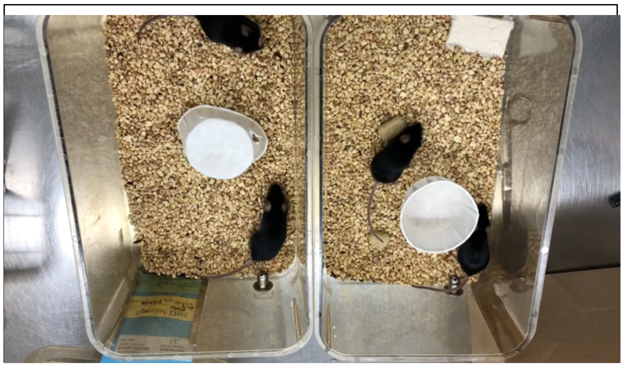

SUPPLEMENTAL VIDEO S3. Mouse activity at 3 days of KA treatment in control and PLX3397-treated mice. Wildtype mice were exposed to either control (left) or PLX3397 (right) chow for 7 days and then treated with KA. This movie is collected at 3 days after KA-induced status epilepticus and PLX3397-treated mice are not as active in exploring their cage The movie is 31 seconds long.

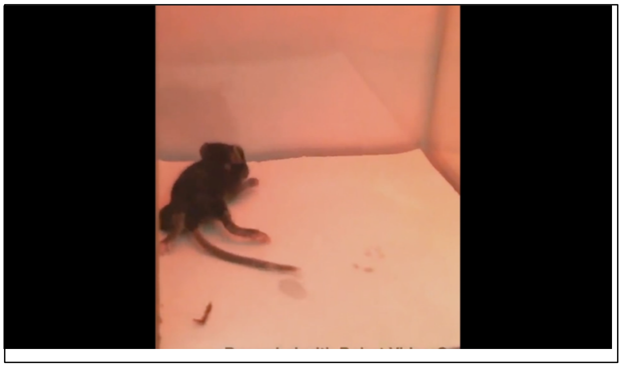

SUPPLEMENTAL VIDEO S4. Neonatal mouse undergoing febrile status epilepticus. Neonatal p14 mouse exposed to hyperthermia showing whole body convulsions. The movie is 1 minute long.
